# Supplementary material for: Resolving the full spectrum of human genome variation using Linked-Reads
Source: Genome Res. 2019 Apr;29(4):635–45. doi: 10.1101/gr.234443.118 (PMC6442396; doi:10.1101/gr.234443.118)
Supplement: Supplemental Material [file supp_gr.234443.118_Supplemental_longranger-2.2.zip › longranger-2.2.tar.bz2!/lr2.2-public/lib/stan/cmdstan/stan/doc/api/html/agrad_2fwd_2matrix_2mdivide__right_8hpp_source.html]

Stan: src/stan/agrad/fwd/matrix/mdivide\_right.hpp Source File


|  |  |
| --- | --- |
|  | Stan  2.5.0  probability, sampling & optimization |


- Main Page
- Related Pages
- Namespaces
- Classes
- Files

- File List
- File Members

All Classes Namespaces Files Functions Variables Typedefs Enumerations Enumerator Friends Macros Pages

- src
- stan
- agrad
- fwd
- matrix

mdivide\_right.hpp

Go to the documentation of this file.

1 #ifndef STAN\_\_AGRAD\_\_FWD\_\_MATRIX\_\_COLUMNS\_MDIVIDE\_RIGHT\_HPP

2 #define STAN\_\_AGRAD\_\_FWD\_\_MATRIX\_\_COLUMNS\_MDIVIDE\_RIGHT\_HPP

3

4 #include <vector>

5 #include <stan/math/matrix/Eigen.hpp>

6 #include <stan/math/matrix/typedefs.hpp>

7 #include <stan/math/matrix/mdivide\_right.hpp>

8 #include <stan/math/error\_handling/matrix/check\_multiplicable.hpp>

9 #include <stan/math/error\_handling/matrix/check\_square.hpp>

10 #include <stan/agrad/fwd/fvar.hpp>

11 #include <stan/agrad/fwd/matrix/typedefs.hpp>

12 #include <stan/agrad/fwd/matrix/inverse.hpp>

13 #include <stan/agrad/fwd/matrix/multiply.hpp>

14 #include <stan/math/matrix/multiply.hpp>

15 #include <stan/agrad/fwd/matrix/to\_fvar.hpp>

16 #include <stan/math/matrix/inverse.hpp>

17 #include <stan/agrad/fwd/matrix/inverse.hpp>

18 #include <stan/math/matrix/mdivide\_right.hpp>

19

20 namespace stan {

21  namespace agrad {

22

23  template <typename T, int R1,int C1,int R2,int C2>

24  inline

25  Eigen::Matrix<fvar<T>,R1,C2>

26  mdivide\_right(const Eigen::Matrix<fvar<T>,R1,C1> &A,

27  const Eigen::Matrix<fvar<T>,R2,C2> &b) {

28

29  using stan::math::multiply;

30  using stan::math::mdivide\_right;

31  stan::math::check\_square("mdivide\_right(%1%)",b,"b",(double\*)0);

32  stan::math::check\_multiplicable("mdivide\_right(%1%)",A,"A",

33  b,"b",(double\*)0);

34

35  Eigen::Matrix<T,R1,C2> A\_mult\_inv\_b(A.rows(),b.cols());

36  Eigen::Matrix<T,R1,C2> deriv\_A\_mult\_inv\_b(A.rows(),b.cols());

37  Eigen::Matrix<T,R2,C2> deriv\_b\_mult\_inv\_b(b.rows(),b.cols());

38  Eigen::Matrix<T,R1,C1> val\_A(A.rows(),A.cols());

39  Eigen::Matrix<T,R1,C1> deriv\_A(A.rows(),A.cols());

40  Eigen::Matrix<T,R2,C2> val\_b(b.rows(),b.cols());

41  Eigen::Matrix<T,R2,C2> deriv\_b(b.rows(),b.cols());

42

43  for (int j = 0; j < A.cols(); j++) {

44  for(int i = 0; i < A.rows(); i++) {

45  val\_A(i,j) = A(i,j).val\_;

46  deriv\_A(i,j) = A(i,j).d\_;

47  }

48  }

49

50  for (int j = 0; j < b.cols(); j++) {

51  for(int i = 0; i < b.rows(); i++) {

52  val\_b(i,j) = b(i,j).val\_;

53  deriv\_b(i,j) = b(i,j).d\_;

54  }

55  }

56

57  A\_mult\_inv\_b = mdivide\_right(val\_A, val\_b);

58  deriv\_A\_mult\_inv\_b = mdivide\_right(deriv\_A, val\_b);

59  deriv\_b\_mult\_inv\_b = mdivide\_right(deriv\_b, val\_b);

60

61  Eigen::Matrix<T,R1,C2> deriv(A.rows(), b.cols());

62  deriv = deriv\_A\_mult\_inv\_b - multiply(A\_mult\_inv\_b, deriv\_b\_mult\_inv\_b);

63

64  return stan::agrad::to\_fvar(A\_mult\_inv\_b, deriv);

65  }

66

67  template <typename T, int R1,int C1,int R2,int C2>

68  inline

69  Eigen::Matrix<fvar<T>,R1,C2>

70  mdivide\_right(const Eigen::Matrix<fvar<T>,R1,C1> &A,

71  const Eigen::Matrix<double,R2,C2> &b) {

72

73  using stan::math::multiply;

74  using stan::math::mdivide\_right;

75  stan::math::check\_square("mdivide\_right(%1%)",b,"b",(double\*)0);

76  stan::math::check\_multiplicable("mdivide\_right(%1%)",A,"A",

77  b,"b",(double\*)0);

78

79  Eigen::Matrix<T,R2,C2> deriv\_b\_mult\_inv\_b(b.rows(),b.cols());

80  Eigen::Matrix<T,R1,C1> val\_A(A.rows(),A.cols());

81  Eigen::Matrix<T,R1,C1> deriv\_A(A.rows(),A.cols());

82

83  for (int j = 0; j < A.cols(); j++) {

84  for(int i = 0; i < A.rows(); i++) {

85  val\_A(i,j) = A(i,j).val\_;

86  deriv\_A(i,j) = A(i,j).d\_;

87  }

88  }

89

90  return stan::agrad::to\_fvar(mdivide\_right(val\_A, b),

91  mdivide\_right(deriv\_A, b));

92  }

93

94  template <typename T, int R1,int C1,int R2,int C2>

95  inline

96  Eigen::Matrix<fvar<T>,R1,C2>

97  mdivide\_right(const Eigen::Matrix<double,R1,C1> &A,

98  const Eigen::Matrix<fvar<T>,R2,C2> &b) {

99

100  using stan::math::multiply;

101  using stan::math::mdivide\_right;

102  stan::math::check\_square("mdivide\_right(%1%)",b,"b",(double\*)0);

103  stan::math::check\_multiplicable("mdivide\_right(%1%)",A,"A",

104  b,"b",(double\*)0);

105  Eigen::Matrix<T,R1,C2>

106  A\_mult\_inv\_b(A.rows(),b.cols());

107  Eigen::Matrix<T,R2,C2> deriv\_b\_mult\_inv\_b(b.rows(),b.cols());

108  Eigen::Matrix<T,R2,C2> val\_b(b.rows(),b.cols());

109  Eigen::Matrix<T,R2,C2> deriv\_b(b.rows(),b.cols());

110

111  for (int j = 0; j < b.cols(); j++) {

112  for(int i = 0; i < b.rows(); i++) {

113  val\_b(i,j) = b(i,j).val\_;

114  deriv\_b(i,j) = b(i,j).d\_;

115  }

116  }

117

118  A\_mult\_inv\_b = mdivide\_right(A, val\_b);

119  deriv\_b\_mult\_inv\_b = mdivide\_right(deriv\_b, val\_b);

120

121  Eigen::Matrix<T,R1,C2>

122  deriv(A.rows(), b.cols());

123  deriv = -multiply(A\_mult\_inv\_b, deriv\_b\_mult\_inv\_b);

124

125  return stan::agrad::to\_fvar(A\_mult\_inv\_b, deriv);

126  }

127  }

128 }

129 #endif

stan::agrad::mdivide\_right

Eigen::Matrix< fvar< T >, R1, C2 > mdivide\_right(const Eigen::Matrix< fvar< T >, R1, C1 > &A, const Eigen::Matrix< fvar< T >, R2, C2 > &b)

**Definition:** mdivide\_right.hpp:26

mdivide\_right.hpp

stan::math::mdivide\_right

Eigen::Matrix< typename boost::math::tools::promote\_args< T1, T2 >::type, R1, C2 > mdivide\_right(const Eigen::Matrix< T1, R1, C1 > &b, const Eigen::Matrix< T2, R2, C2 > &A)

Returns the solution of the system Ax=b.

**Definition:** mdivide\_right.hpp:25

check\_multiplicable.hpp

inverse.hpp

typedefs.hpp

stan::agrad::fvar

**Definition:** fvar.hpp:13

stan::agrad::to\_fvar

fvar< T > to\_fvar(const T &x)

**Definition:** to\_fvar.hpp:17

typedefs.hpp

stan::agrad::multiply

Eigen::Matrix< fvar< T >, R1, C1 > multiply(const Eigen::Matrix< fvar< T >, R1, C1 > &m, const fvar< T > &c)

**Definition:** multiply.hpp:21

stan::math::check\_multiplicable

bool check\_multiplicable(const char \*function, const T1 &y1, const char \*name1, const T2 &y2, const char \*name2, T\_result \*result)

**Definition:** check\_multiplicable.hpp:15

fvar.hpp

inverse.hpp

Eigen.hpp

to\_fvar.hpp

multiply.hpp

stan::math::multiply

boost::enable\_if\_c< boost::is\_arithmetic< T >::value, Eigen::Matrix< double, R, C > >::type multiply(const Eigen::Matrix< double, R, C > &m, T c)

Return specified matrix multiplied by specified scalar.

**Definition:** multiply.hpp:25

multiply.hpp

stan::math::check\_square

bool check\_square(const char \*function, const Eigen::Matrix< T\_y, Eigen::Dynamic, Eigen::Dynamic > &y, const char \*name, T\_result \*result)

Return true if the specified matrix is square.

**Definition:** check\_square.hpp:24

check\_square.hpp


---

[ Stan Home Page ]

*© 2011–2014,
Stan Development Team.*
